# Open and closed economies as possible alternative strategies to resource heterogeneity in ants

**DOI:** 10.1242/bio.061976

**Published:** 2025-05-27

**Authors:** Kazuki Tsuji, Aye T. Win

**Affiliations:** ^1^ Department of Agro-Environmental Sciences, Faculty of Agriculture, University of the Ryukyus, Nishihara, Okinawa 903-0213, Japan; ^2^ Division of Rural Communities and Environmental Sciences, The United Graduate School of Agricultural Sciences, Kagoshima University, Korimoto 1-21-24, Kagoshima 890-8580, Japan

**Keywords:** Decentralized society, Monodomy, Polydomy, Resource redistribution, Nutrition, Biological invasion

## Abstract

Ant colonies have either a single nest (monodomy) or multiple nests (polydomy). A challenge is to explain their adaptive significance, specifying costs and benefits of each colony type. An explanation for ant polydomy is adaptation to spatially heterogeneous environments. With polydomy a colony can exchange complementary nutrition among nests within the entire colony occupying a wide territory. We tested this resource redistribution hypothesis using two closely related species, i.e. the polydomous ant *Pheidole megacephala* and the monodomous ant *Pheidole noda.* We put each colony in an artificially polydomous setting with two nests connected by tubes. We provided liquid food lacking protein to one nest and that lacking carbohydrates to the other nest. *P. megacephala* almost totally failed to produce brood when the connecting tubes were clipped, whereas it improved reproductive performance when the tubes were open. In marked contrast, *P. noda* managed to maintain high performance for a long period even when only nutritionally biased food was provided, most likely by relying on stored provisions that compensated for the missing nutrients. Based on these data, we propose the hypothesis that ant polydomy is an open economic strategy to counter heterogeneity in resource distribution ‘spatially’ by trading between nests and extending the resource searching area, whereas monodomy may be a closed economic strategy to cope with resource heterogeneity ‘temporally’ by withstanding food-depressed periods with stored nutrition and by efficient utilization of frugal diets. More empirical data in other ant taxa are necessary to test generality of this idea.

## Teaser

**Summary:** Free trading and protectionism are opposite policies in human economies. Similar alternative strategies to cope with resource heterogeneity were suggested in ants, related to the nest number per colony.

## INTRODUCTION

Many animals performing parental care forage from a central place, bringing food collected in the surrounding environments to the nest to feed brood. The spatial location of the nest in the habitat can strongly influence reproductive performance in those animals, because it should determine the accessibility to food and other resources. Social insects are typical of such central-place foragers (Hölldobler and Lumsden, 1980) in which a nest is owned by a colony – a group of cooperatively breeding individuals. Ant colonies can be categorized as monodomous, in which a colony occupies a single nest, or polydomous, in which a colony possesses multiple spatially separated nests among which individuals can move (Hölldobler and Wilson, 1990; Sundström, 2021). The adaptive significance of those colony structures, specifying costs and benefits of each colony type, has been challenged (Debout et al., 2007; Robinson, 2014; Burns et al., 2019; Rodrigues et al., 2022).

Although many factors have been proposed to affect the colony-type-associated costs and benefits, such as nest homeostasis, risk avoidance, information transmission, resource distribution, per worker colony efficiency, and genetic structure of individuals and colonies (Debout et al., 2007; Kramer et al., 2012; Stroeymeyt et al., 2017; Burns et al., 2019), hypothetical driving forces favoring polydomy rather than monodomy can be divided into two categories: (1) intracolony evolutionary conflict, such that some workers leave the nest to avoid queen control, leading to polydomy (e.g. Snyder and Herbers, 1991; Giehr et al., 2020) and (2) colony-level adaptation to environments (e.g. Cook et al., 2013; Burns et al., 2019). In this study, we focus on the latter view.

Polydomous ants seem to adopt a dispersed central-place foraging strategy in which resources collected by a nest are later redistributed to other nests in the colony. Burns et al. (2019) discussed by using a space-explicit simulation model that this strategy can improve the colony-level efficiency in some environments, in particular, when foraging costs are high, nest size is limited, resources are clustered, and nests are frequently disturbed (see also Cook et al., 2013 but see Schmolke 2009), whereas the centralized strategy (monodomy) is advantageous when resource availability fluctuates and nest size is limited. Empirical evidence supporting this colony-level benefit hypothesis is yet scarce and seemingly anecdotal. For example, in *Temnothorax nylanderi*, polydomy facilitates quicker resource retrieval thorough inter-nest information transmission (Stroeymeyt et al., 2017). In *Formica lugubris* colonies appear to maintain productivity by flexibly altering their trail network among polydomous nests when their access to the main food source is blocked (Burns et al., 2021).

The scope of the model by Burns et al. (2019) may be limited, because they assume only one kind of food resource. Many real ants are omnivores, feeding on multiple complementary food sources. Typically, the carbohydrates required for adult activity are obtained from plant nectar and insect honeydew, while proteins required for larval development are obtained from hunting. Holway and Case (2000) discussed ant polydomy in this context and experimentally showed that the Argentine ant, *Linepithema humile*, counters the spatially heterogeneous distribution of foods through redistribution of foods of complementary nutrition, prey and nectar, within the polydomous colony. This is a parallel phenomenon to the physiological integration in clonal plants in which ramets are interconnected by a root or a stolon to exchange complementary resources, thereby allowing the plants to adapt to spatially heterogeneous environments (Shumway, 1995; Roiloa et al., 2010; Xu et al., 2010). Experimental evidence of enhancement of reproductive performance by such nest-to-nest resource redistribution in polydomous ants is, however, lacking except for the Argentine ant case so far. Furthermore, it is unknown whether monodomous ants are less adapted to heterogeneous environments or cope in some way without resorting to polydomy.

**Fig. 1.**
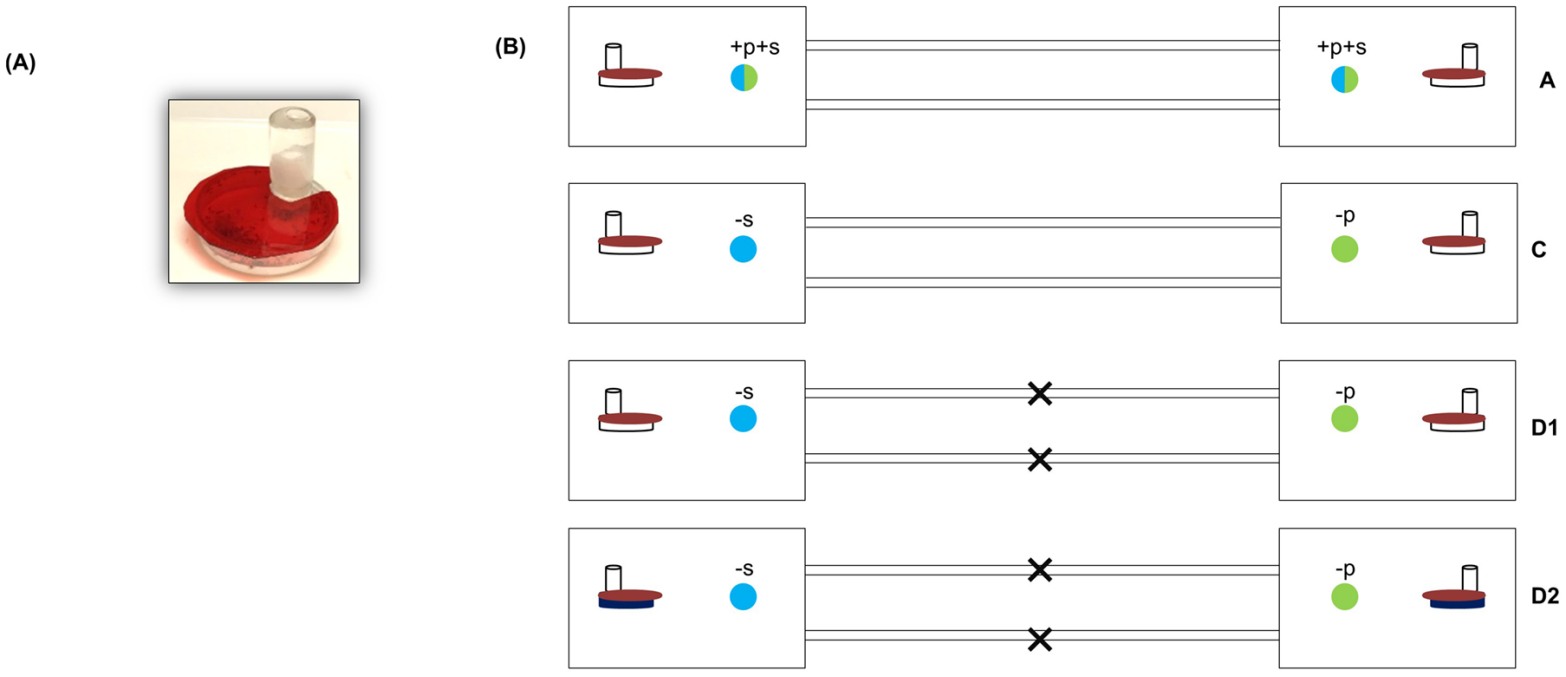
**(A) An artificial ant nest and (B) schematic illustration of the experimental design and treatments of the resource redistribution experiment.** In Treatment A (control provided foods with all nutrition) and Treatment C (provided with complementary foods, protein-lacking food to one side and carbohydrate-lacking food to the other), two nests were connected by tubes, except at feeding time (when the tubes were clipped). In Treatments D1 (negative control 1: equal queen/worker ratio to Treatments A and C) and D2 (negative control 2: each side had equal number of workers as in entire A and entire C), foods were provisioned in the same way as in Treatment C, but the two nests were always disconnected by clipping the tubes. Two experimental arenas consisting of nest chambers connected by two plastic tubes were provided with different types of food (shown in colored circles).

In this study, we investigated responses to spatially heterogeneous resource distribution in two species of ant in genus *Pheidole* (Hymenoptera: subfamily Myrmicinae); one is monodomous *Pheidole noda* and the other is polydomous *Pheidole megacephala*. We examined the occurrence of resource redistribution among nests in an experimentally settled polydomous colony in the laboratory (Fig. 1A,B), an approach similar to Holway and Case (2000). When each nest was provided with complimentary foods (lacking protein or lacking carbohydrates), we tested whether nest-connected colonies had superior colony growth and worker survival than unconnected ones. We observed whether there was any differential performance under resource heterogeneity between the two species with different colony types. This relates to a more general question. We hypothesize two alternative types of ant economy. One is an open economy in which the redistribution of resources among nests contributes to the fitness and/or inclusive fitness of individual colony members, and the other is a closed economy in which the securing and distribution of resources are completed within members of an individual nest. Note that *P. noda* is monodomous and therefore has a closed economy in nature. We predicted that *P. noda* would not be able to take advantage of the experimentally provided polydomous nest structure in its behavior to cope with environmental heterogeneity, because it may lack the behavioral adaptation to polydomy.

## RESULTS

### Resource redistribution experiment: brood production

**Fig. 2.**
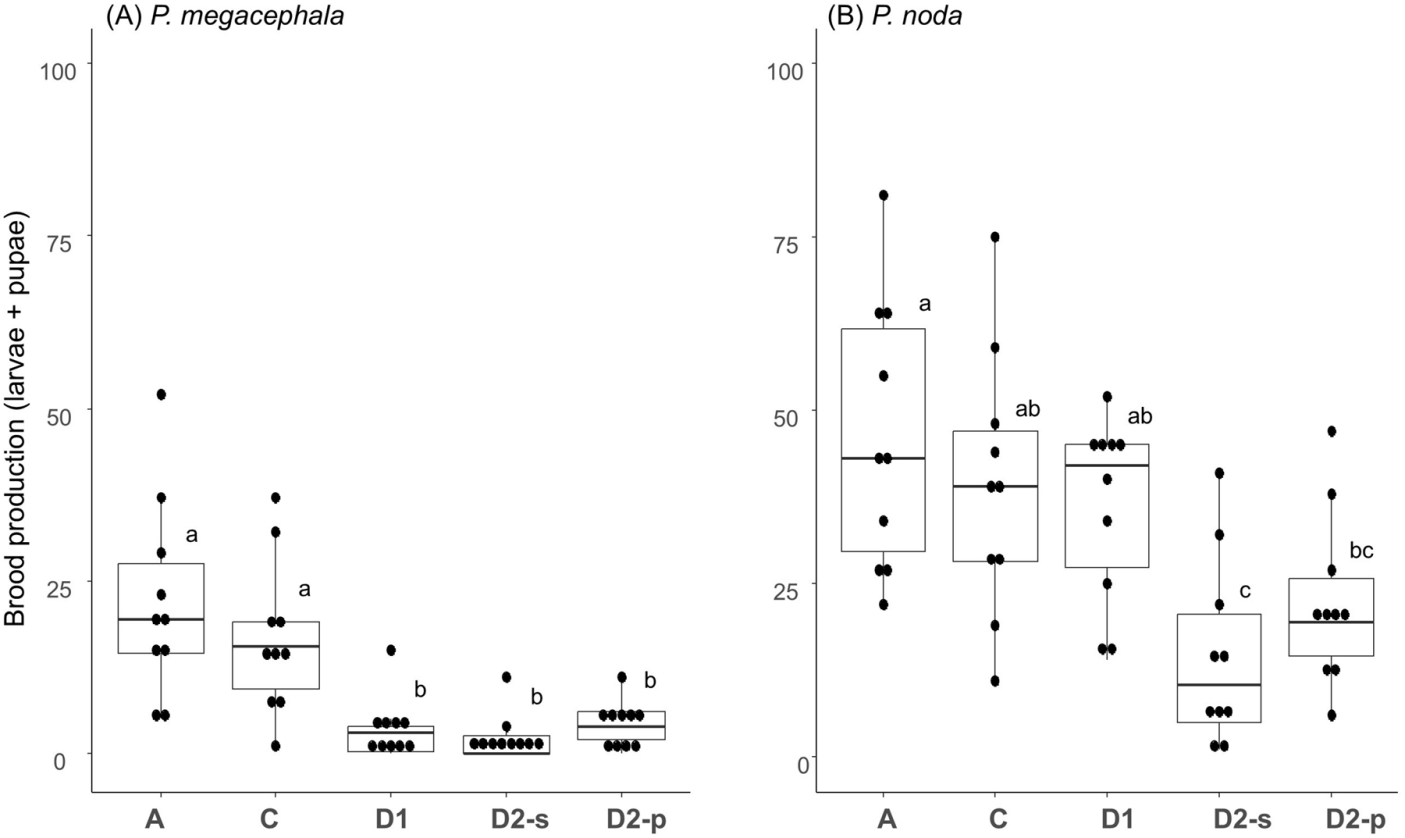
**Box-plot results of the number of total broods in the treatments.** Treatment A (all nutrition provided positive control), C (connected-nests treatment), D1 (equal queen:worker ratio negative control), D2-s (carbohydrate-lacking nest in the equal worker number negative control), and D2-p (protein-lacking nest in the equal worker number negative control). (A) Polydomous species, *P. megacephala*, and (B) monandrous species, *P. noda*. The upper and lower sides of the box represent the interquartile range, and the dark line is the median. The bars extending from the box indicate the highest and lowest values excluding outliers, which are presented as dark dots. Different letters (a and b) indicate statistical differences in post hoc comparison (Wilcoxon signed rank test at *P<*0.05 after Bonferroni correction in *P. megacephala*, and pairwise *t*-test at *P<*0.05 after Bonferroni correction in *P. noda*). We also tested the statistical significance, effect size, and 95% confidence interval of the entire models [A: Friedman test, χ^2^_(4)_ =22.741, *P*=0.0001, W=0.569, CI=0.460, 0.770; B: one-way repeated measure ANOVA, *F*_(4, 36)_=8.985, *P*<0.001, η_p_^2^=0.500, CI=0.270, 1.000].

In the polydomous species, *P. megacephala*, when we provisioned a protein-lacking food to one nest and a carbohydrate-lacking food to the other nest in Treatment C (the nest-connected treatment), colonies produced as much brood as the treatment in which food containing all nutrients was provisioned to both nests (Treatment A; Fig. 2A). When the two complementary-food-provisioned nests were disconnected by clipping the tubes (Treatments D1 and D2), brood production was markedly and significantly decreased, compared to Treatment A (Fig. 2A). This result supports the resource redistribution hypothesis. In Treatment C, brood appeared to be located in the nest where protein was provisioned, which was, however, not statistically significant (V=44, *P*=0.10, Table 1).

**Table 1.** Position of brood and workers at the end of the resource redistribution experiment

Species	Treatment A			Treatment C		
	Complete food Nest 1	Complete food Nest 2	Statistical test	Protein-provisioned nest	Sugar-provisioned nest	Statistical test
Brood						
*P. megacephala*	21.50±7.36	13.10±4.02	V=38, *P=*0.32	25.70±7.16	9.80±2.85	V=43, *P*=0.13
*P. noda*	41.43±11.98	17.86±8.86	t_6_=1.25, *P*=0.26	23.43±12.27	25.57±6.75	V= 11, *P*=0.92
Workers						
*P. megacephala*	36.80±4.71	39.10±7.19	t_9_=−0.36 *P*=0.73	63.80±7.85	89.00±10.38	t_9_=−1.76 *P*=0.11
*P. noda*	37.57±8.29	19.57±7.39	V=25, *P*=0.08	22.86±7.77	29.14±9.93	t_6_=−0.48, *P=*0.65

Mean±s.e. numbers of brood (eggs, larvae, and pupae) and workers found in each nest of connected Treatments A and C in polydomous *P. megacephala* and monodomous *P. noda* at the end of the experiment. In Treatment A, both nests were provisioned with the food containing all nutrients, so we randomly assigned the nest ID as Nest 1 or Nest 2.

In the monodomous species, *P*. *noda*, unexpectedly, Treatments A, C and D1 (a disconnected treatment) showed a similar high reproductive performance (Fig. 2B). The high performance of Treatment D1 suggests that even when only a nutritionally unbalanced food, protein-lacking or carbohydrate-lacking, was available, *P. noda* colonies were able to continue to produce brood for 30 days at a level similar to colonies provisioned with food containing all nutrients. Interestingly, the other disconnected-nests treatment (D2-s and D2-p) showed significantly lower brood production than Treatment A (Fig. 2B). The difference between Treatments D1 and D2 is in the queen/worker ratio, meaning that a nutritional imbalance harmed colony performance when the queen/worker ratio was relatively low. On average, the brood was located almost equally in the two connected nests in Treatments A and C (Table 1).

### Resource redistribution experiment: worker survival

**Fig. 3.**
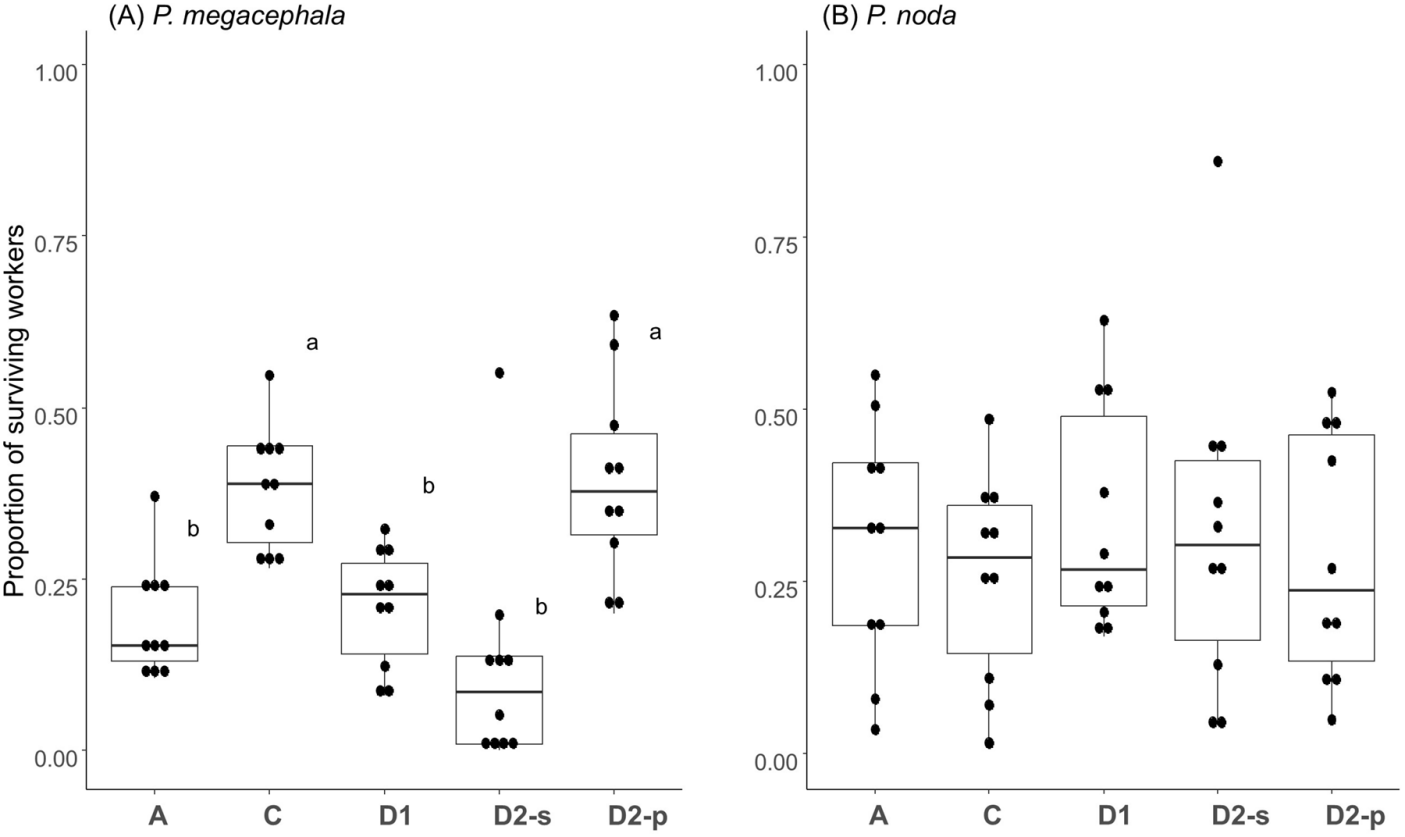
**Box-plot results of the proportion of surviving workers in the treatments.** Treatment A (all nutrition provided positive control), C (connected-nests treatment), D1 (equal queen/worker ratio negative control), D2-s (carbohydrate-lacking nest in the equal worker number negative control), and D2-p (protein-lacking nest in the equal worker number negative control). (A) Polydomous species, *P. megacephala*, and (B) monodomous species, *P. noda*. The upper and lower sides of the box represent the interquartile range, and the dark line is the median. The bars extending from the box indicate the highest and lowest values excluding outliers, which are presented as dark dots. Different letters (a and b) indicate statistical differences in post hoc comparison (pairwise *t*-test at *P<*0.05 after Bonferroni correction). We tested statistical significance, effect size, and 95% confidence interval of entire models [A: one-way repeated measure ANOVA, *F*_(4, 36)_=12.070, *P*<0.001, η_p_^2^=0.570, CI=0.360, 1.000; B: one-way repeated measure ANOVA, *F*_(4, 36)_=0.368, *P*=0. 0.830, η_p_^2^=0.040, CI=0.000, 1.000].

In the polydomous species, *P. megacephala*, the protein-lacking side of the disconnected treatment (D2-p) showed the highest worker survival (post hoc test, *P*<0.05) among the five treatments. Among the other treatments, Treatment C with connected nests showed higher performance (post hoc test, *P<*0.05) than those of Treatments A, D1, and D2-s (Fig. 3A). At the end of the experiment, almost equal numbers of workers were found in both nests in the connected Treatments A and C (Table 1).

In the monodomous species, *P. noda*, however, we found almost no difference in worker survival among treatments (Fig. 3B). At the end of the experiment, nearly equal numbers of workers were found in both nests of connected Treatment C (Table 1).

### Detection of trophallaxis between workers that first belonged to different nests in *P. megacephala*

The number of white-paint-marked workers that contained Rhodamine B color found in Nest 1 and Nest 2 were as follows: Colony 1 had 4 color-dyed workers out of 91 workers in Nest 1 and 0 out of 71 in Nest 2. Colony 2 had 12 out of 115 in Nest 1 and 4 out of 33 in Nest 2. Colony 3 had 22 out of 85 in Nest 1 and 24 out of 64 in Nest 2. In all three colonies, we detected the color in a portion of marked workers that did not directly consume the dyed food on the first day suggesting food sharing via worker–worker trophallaxis across nests. On the second day, we also found some marked workers in Nest 2, which indicates that some workers actually moved between nests.

### A long-term brood production in *P. noda* colonies when fed only sugar

Over 8 weeks, regardless of the food provisioning treatments, all *P. noda* colonies contained some brood. At the end of the experimental period, there was no significant difference between treatments in the composition of brood contained in the nests or in the number of surviving workers (Table 2).

**Table 2.** Colony performance in *P. noda* in terms of the numbers of brood and surviving workers after 8 weeks

Type	All nutrition treatment (mean±s.e.)	Sugar-only treatment (mean±s.e.)	Statistical test
Eggs	11.00±2.57	9.89±3.27	*t*=0.27, *P*=0.79
Larvae	18.78±3.34	15.11±4.16	*t*=0.69, *P*=0.50
Pupae	2.89±0.84	3.44±1.77	W=45.5, *P*=0.69
Total brood	32.67±6.17	28.44±8.18	*t*=0.41, *P*=0.69
Workers	37.22±9.54	33.22±9.03	*t*=0.30, *P*=0.76

## DISCUSSION

We have confirmed that *P. megacephala* exchanged limited resources between nests and thereby improved their reproductive performance (i.e. brood production) to a level comparable with nests provisioned with nutritionally balanced food. The experiment with colored food directly showed evidence for such food exchange between nests through worker–worker trophallaxis. Our results support the view of Holway and Case (2000) who first showed such sharing of complementary nutrition between nests in the Argentine ant and hypothesized that other polydomous invasive ants, including *P. megacephala*, would exhibit this behavior as well. Note that in our experiment the foods provisioned were perfectly complementary – one lacking carbohydrate and the other lacking protein – because we used artificial proteins, which differed from the previous study that used real insect prey as the protein source. *P. megacephala* may adopt an open-economy strategy to counter heterogeneous resource distribution by sharing between nests and extending the resource searching area.

We also found an unexpected result in the monodomous ant, *P. noda*. Colonies sustained reproductive performance for 30 days even under biased food conditions. Quite unexpectedly, the colony performance in a control treatment (D1), where nests provisioned with complementary foods were totally disconnected, were comparable with that of Treatment A (the all nutrition-provisioned treatment). Because of this result, it could not be determined whether the fact that performance of Treatment C was similar to that of Treatment A in *P. noda* was due to resource redistribution. We consider, however, that resource redistribution is unlikely since this ant is monodomous in the wild. Instead, a hypothetical mechanism is nutrition storage. We infer that *P. noda* queens store nutrition in their bodies – perhaps in fat bodies and the crop – that can be redistributed to colony members when foraging becomes temporally less successful. This interpretation comes from the observation that the reproductive performance of the other disconnected control treatment (D2) dramatically declined. The worker:queen ratio of Treatment D2 was twice as large as that of Treatment D1. We infer that the putative queen-stored nutrition might be insufficient to support the many workers in D2 colonies.

The concentration of protein in foods (in the whole colony for Treatments A and C and in the nest for Treatments D1-D2) was negatively correlated with worker survival in the polydomous *P. megacephala* (Fig. 3A). An additional experiment provisioning foods with various protein concentrations confirmed this pattern (Fig. S2). The pattern in *P. megacephala* is in line with what is known in another omnivorous ant (Dussutour and Simpson, 2009; Cook et al., 2010; Lihoreau et al., 2015) and in insects in general (Simpson and Raubenheimer, 2012), namely that protein is indispensable to larval growth but toxic to adults. Under this scenario, the division of labor among workers in foraging items can be beneficial in terms of worker survival by limiting the number of workers that are exposed to protein-rich foods. Indeed, when protein and carbohydrate were provisioned separately (Treatment C), worker survival was improved as compared with the provision of food containing all nutrients to both nests (Treatment A; Fig. 3A).

Notably, worker survival of *P. noda* was not associated with the protein concentration of food (Fig. 3B). The remarkable protein ‘tolerance’ in *P. noda* might be related to the species' natural food menu. On the island of Okinawa, Japan, *P. noda* is almost never observed to forage homopteran honeydew (Tanaka et al., 2011). This suggests that *P. noda* does not strongly rely on sugary carbohydrates for energy in the field. Instead, they hunt or scavenge small animals and might obtain energy from those foods presumably through metabolizing lipid and protein. Furthermore, in captive conditions *P. noda* workers frequently consume nestmates' corpses, whereas *P. megacephala* workers do not (K. Tsuji and A.T. Win, personal observation). This may suggest that *P. noda* is a good resource recycler, as discussed in other ants who rely on extreme diets (Bisch et al., 2018).

In *Pheidole*, major workers can function as repletes that regurgitate liquid nutrition stored in the crop and share it with other colony members when necessary (see Introduction). Therefore, admittedly, similar experiments using colonies containing major workers are necessary to see their tolerance to unbalanced nutrition provisioning under more natural colony composition.

Although our experiments were performed in artificial environments using small colonies without major workers, some implication might be possible. Because of its monodomous nature, *P. noda* may employ a closed-economy strategy to deal with resource heterogeneity temporally by withstanding food-depressed periods with stored nutrition and by efficient utilization of frugal diets via resource recycling. Note that *P. noda* colonies continued to raise larvae for at least 8 weeks when only sugar was provided as food. This view is in line with the theoretical prediction by Burns et al. (2019) that temporally heterogeneous resource distribution can favor monodomy in some situations. In contrast, polydomous *P. megacephala* may adopt an open-economy strategy to counter such heterogeneity spatially by extending the area of resource searching through the physiological integration of nests. We cannot yet generalize this as a strategic difference between monodomous and polydomous ants. Recent empirical studies suggest that inter-nest resource sharing in polydomous ants may not be so free as previously thought, for instance in *Formica* (Helanterä, 2022) and *Solenopsis invicta* (Kjeldgaard et al., 2022) resource sharing is seemingly spatially restricted. More empirical data are needed, especially in other ant taxa.

## MATERIALS AND METHODS

### Study species

We used two ant species in the genus *Pheidole* (Hymenoptera: Myrmicinae) collected at various locations on the main island of Okinawa, Japan, during 2016–2024. The big-headed ant, *P. megacephala*, is an exotic species that is polydomous and polygynous with a unicolonial population structure. It is regarded as a harmful species and is listed in the IUCN's blacklist of 100 world-wide invasive species (Lowe et al., 2001). *Pheidole noda* is native to Okinawa. This ant is also polygynous, but queens independently found colonies and the workers from nearest neighbor nests are strongly hostile, indicating monodomy (K. Tsuji and A.T. Win, personal observation). These two ants fit our comparative aims, because they are both common on Okinawa and minor workers have similar body sizes (*P. megacephala*, ca. 2 mm; *P. noda*, ca. 3 mm). The main habitats of *P. megacephala* are disturbed environments such as urban parks and beachside sandy habitats, whereas that of *P. noda* is the forest floor. However, the two species coexist on forest edges and in wooded parks (Yamauchi and Ogata, 1995; Katayama and Tsuji, 2010; Tanaka et al., 2011). Furthermore, both species forage making pheromonal trails.

*Pheidole megacephala* and *P. noda* colonies were collected and brought to the laboratory (14:10 h L:D photocycle, 25±1°C, 60–70% R.H.). Each colony contained multiple queens and multiple major workers in addition to 30–2000 minor workers (in *P. noda*) and thousands of minor workers (in *P. megacephala*). Each of these colonies (‘stock colonies’ hereafter) was maintained individually in a large plastic box coated with Fluon (AGC Chemicals, Exton, PA, USA), filled with substrate from the original nest materials (soil, wood debris), and containing several glass test tubes filled with water that were stoppered with a cotton ball. Prior to the start of the experiments, we fed colonies with 10% honey water, chopped mealworms, and various field-collected insects three times a week. Because *P. megacephala* is unicolonial, their colony identities are admittedly uncertain.

### Artificial foods

We used an artificial diet in which the protein source was tryptone, an industrially produced artificial protein (Wako Pure Chemical Industrial Ltd., Osaka, Japan). We examined the suitability of tryptone as food elsewhere (Fig. S1). Previous studies on related topics (Holway and Case, 2000; Dussutour and Simpson, 2008a) used insect prey or hen eggs as the protein source. We avoided this because those animal materials also contain non-protein nutrients, such as lipids, sterols, vitamins, and minerals. By using artificial protein, we were able to change the proportion of protein while keeping the amounts of the other nutrients constant. The artificial foods we used are mixtures of protein, carbohydrate, lipids, vitamins, and minerals. Other than tryptone, sucrose (Kanto Chemical Co. Ltd., Tokyo, Japan) as a carbohydrate source, rapeseed oil (Boso Oil and Fat Co. Ltd., Tokyo, Japan) as a lipid source, Vanderzent vitamin mixture for insects (Sigma-Aldrich, St. Louis, USA) as a vitamin source, and salt (Paradise Plan Co. Ltd., Miyakojima, Japan) as a mineral source were used. Following the recipe of Dusstour and Simpson (2008b), with some modifications, we prepared the food mixture with all nutrients that includes 13 g of carbohydrate, 7 g of protein, 1.2 g of vitamins, 1.2 g of minerals, and 1.4 g of lipid in 100 g of distilled water solution. We also prepared protein-lacking food in which only protein was excluded while keeping concentrations of the other contents in water solution unchanged. Similarly, we prepared carbohydrate-lacking food in which carbohydrate was excluded. Just before provisioning, each food was mixed well by a vortex.

### Resource redistribution experiment

In all experiments, a group of ants (the composition of which is described later) from the same stock colony was housed in nests made of small Petri dishes (55 mm diameter) with a layer of plaster of Paris in the bottom and red cellophane covered lid to provide ‘darkness’ (Fig. 1A). Minor workers and brood carried by the workers were able to enter/exit the nest through four small circular holes (1-mm diameter) on the side of the dish, but the queen was unable to do so. The ant nest was provided with a constant supply of water from a cotton-stoppered water-filled (7 ml) glass vial that was positioned upside down on top of the dish (Fig. 1A). Each nest was placed in a plastic box (23×16.5×9 cm; hereafter, ‘experimental arena’) with Fluon-coated walls to prevent the ants from escaping. All experimental colonies were moved into the nests in the experimental arenas on the day before starting the experiment.

To test the resource redistribution hypothesis, two experimental arenas were connected by two 1-m-long plastic tubes (diameter 10 mm) (Fig. 1B). We set the inter-arena distance to 1 m because in the field we often observed *P. megacephala* nests located within this distance, though it was sometimes farther. We prepared the following three treatments. In Treatment A, both experimental nests were provisioned with the all-nutrition-mixture food. In Treatment C, one nest was provided with the protein-lacking food and the other was provided with the carbohydrate-lacking food. In Treatment D1, the food condition was the same as that of Treatment C, but the tubes connecting the two boxes were always closed by clips so that workers could not move between the nests. In Treatments A and C, the clips were removed except during the time when the foods were provisioned, and the workers were able to freely move between the nests. Every day for 3 h, we supplied to each nest a 1000-μl droplet of the above defined liquid food put on a small plastic Petri dish cover (40 mm diameter), which was placed about 7 cm from the nest entrance.

In each nest of Treatments A, C, and D1, at the beginning we housed one queen and 100 minor workers for *P. noda* and one queen and 200 minor workers for *P. megacephala*. The difference in the minor worker number reflected the difference in the colony size (the majority of *P. noda* colonies contain fewer than 1000 workers, whereas those of unicolonial *P. megacephala* often have thousands of workers) and the body size of minor workers (ca. 3 mm for *P. noda* and ca. 2 mm for *P. megacephala*). The wet biomass of 100 minor workers of *P. noda* (average±s.d. in mg was 55.33±13.47, *N*=6 colonies) was comparable with that of 200 *P. megacephala* minor workers (48.0±2.10, *N*=6). We did not use major workers because in *Pheidole* they are known to serve as repletes (the nutrient storage caste) (Tsuji, 1990; Lachaud et al., 1992; Yang, 2006), which would complicate the experimental design.

We also prepared Treatment D2, in which the tubes connecting the two boxes were always closed by clips and each nest had a single queen and double the number of minor workers (i.e. 200 in *P. noda* and 400 in *P. megacephala*). In Treatment D2, the colony size (worker density) of each disconnected nest was the same as that of the entire colony (the sum of two connected nests) of Treatments A and C, whereas in Treatment D1 the queen:worker ratio of each disconnected nest was comparable to the entire colony of Treatments A and C. In Treatment D2, we compared the performance of each nest (D2-s was provisioned with food lacking carbohydrate and D2-p was provisioned with food lacking protein) to that of total colony performance of Treatments A, C, and D1. This allowed us to control for the number of workers at the beginning.

Throughout the above experiments, we employed a block design, that is, we prepared all treatments (A, C, D1, and D2) once from each stock colony (*n*=10 colonies in each species).

All colonies were allowed to feed for 3 h per day, and then all food dishes were removed from the experimental arenas.

We set the experimental period as 35 days for *P. megacephala* and as 30 days for *P. noda*, which approximately corresponded to the average growth period from egg to pupal stage in each species under our experimental conditions (K. Tsuji and A.T. Win, personal observation). At the end of the experimental period, we counted the numbers of surviving workers and brood (eggs, larvae, and pupae) in each nest for each treatment.

### Detection of trophallaxis between workers that first belonged to different nests in the resource redistribution experiment of *P. megacephala*

To confirm redistribution of food resources between nests in the polydomous ant species directly, we prepared laboratory colonies (*N*=3) of *P. megacephala* for which the initial setup was the same as Treatment C of the resource redistribution experiment. To one nest we provisioned a food lacking protein (Nest 2) and to the other nest a food lacking carbohydrate (Nest 1), as in the main experiment. All workers (*N*=200) housed in Nest 1 were marked with white paint on their abdomens. In this experiment, the food provisioned on the first day to Nest 2 contained 0.3% Rhodamine B (but the food to Nest 1 was undyed). The tubes connecting the two boxes remained closed by clips until finishing this first feeding period. On the second and third days provisioned food did not contain Rhodamine B. After the feeding time on day 3, all marked workers were killed and crushed on a white filter paper in order to detect the color of Rhodamine B.

### Long-term colony performance in *P. noda* colonies when fed only sugar

We obtained evidence that colonies of *P. noda* maintain performance for approximately 1 month even when only biased food resources are available (see Results). Then, we monitored brood production of *P. noda* colonies weekly for 8 weeks when only sucrose was provided as food compared with the provision of food containing all nutrients (the recipe was the same as in the resource redistribution experiment). From each of the stock colonies (*N*=9) we established a pair of two equal-sized sub-colonies with one queen and 200 minor workers; one was assigned as the complete nutrient treatment (the same food as Treatment A) and the other as the sugar-only treatment. Each week we checked for the presence of brood in each colony. At the end of the experiment, we examined the colony contents.

### Statistical analyses

All statistical analyses were performed using the R software, v. 3.0.2 (R Development Core Team, 2013). Brood production performance was evaluated as the total number of brood excluding eggs (i.e. the number of larvae and pupae). We adopted this measurement because some eggs will be consumed as food (trophic eggs) and thus do not develop to adulthood. Before analyses, all data were tested for normal distribution by using the Shapiro–Wilk test and for homogeneity of variances by using Levene's test. In *P. megacephala*, data on the number of brood did not follow a normal distribution, so we used non-parametric Friedman's test. Treatments (A, C, D1, D2-s, D2-p) were regarded as fixed factors and each ant colony was considered a block. A pairwise Wilcoxon signed rank test with post hoc Bonferroni correction was performed to test pairwise differences between treatments. To compare the number of surviving workers among treatments, one-way repeated measure ANOVA with treatments was performed, followed by multiple comparisons using a pairwise *t*-test. In *P. noda*, data of brood production (both total number and that excluding eggs) and the number of surviving workers did not deviate significantly from a normal distribution, therefore we used one-way ANOVA with repeated measures (or blocks). Treatments (A, C, D1, D2-s, D2-p) were considered as fixed factors and each ant colony was considered a block (random). A pairwise *t*-test with post hoc Bonferroni correction was performed to test pairwise differences between treatments. We also analyzed the numbers of brood and surviving workers found in each nest in the connected Treatments A and C at the end of the experiment to assess whether brood and workers were more likely to be located in the nest closer to the protein source in Treatment C. If the data did not significantly deviate from a normal distribution, a paired *t*-test was performed. A Wilcoxon signed rank test was used if the deviation of data from normality was detected.

### Compliance with regulations of animal experiments

We complied with regulations and guidelines of animal care and use in Japan and the University of the Ryukyus.

## Supplementary Material

10.1242/biolopen.061976_sup1 Supplementary information
