# Supplementary material for: Open and closed economies as possible alternative strategies to resource heterogeneity in ants
Source: Biol Open. 2025 May 27;14(5):bio061976. doi: 10.1242/bio.061976 (PMC12147459; doi:10.1242/bio.061976)
Supplement: Supplementary information [file biolopen-14-061976-s1.pdf]

Biology Open • Supplementary information

Suitability of tryptone as a pure protein source for the growth of *Pheidole megacephala* and *P. noda*

To choose an appropriate artificial protein, we examined brood production of *Pheidole megacephala* and *P. noda* by provisioning two artificial proteins, tryptone (T) and lactalbumin hydrolysate (L), which were compared with the control treatment in which *Tenebrio molitor* mealworms (M) were used as the protein source. We prepared 100 g distilled water solution that included, in addition to 5% of one of the two proteins or crushed dry mealworm, 10% sucrose as the carbohydrate source, 1% rapeseed oil as the lipid source, 1% Vanderzent vitamin mixture for insects (Sigma-Aldrich) as the vitamin source, 1% salt (Paradise Plan Co. Ltd.) as the mineral source. We gave ants one of those diets for 34 consecutive days and then compared reproductive performance (N = 5 colonies for each species). At the beginning, each colony contained one queen and workers (200 minor workers for *P. megacephala*; and 100 minor workers for *P. noda*). Each was kept in a plastic box (16 × 23 × 9 cm) containing a nest (the same nest design was used as in main experiments), with side-walls coated with Fluon to prevent ants from escaping. Each day for 3 h we provisioned 500 µl of food placed on a 3-cm-diameter Petri dish about 5 cm from the nest entrance. On day 35 we counted the total number of brood (larvae + pupae) as a brood production measure. Eggs were not included in this measure, because some eggs are consumed as trophic eggs and do not grow.

As to the total number of brood we used the raw data in *P. noda*, whereas in *P. megacephala* Shapiro-Wilk test detected significant deviation from normal distribution thus data were square root-transformed before analysis. A repeated measure ANOVA (colony is the block) detected no statistically significant difference in the total number of brood among the three types of foods in *P. megacephala* ($F_{(2, 8)}$ = 1.34, p = 0.383, Fig. S1a). A statistically significant difference was found among the tree types of foods in *P. noda* ($F_{(2, 8)}$ = 7.02, p = 0.017, Figure S1b). However, this was because lactalbumin hydrolysate (L) performed

significantly better than the natural food, mealworms (M). There was no significant difference between mealworms and tryptone. Therefore, we chose tryptone to use in the main experiments.

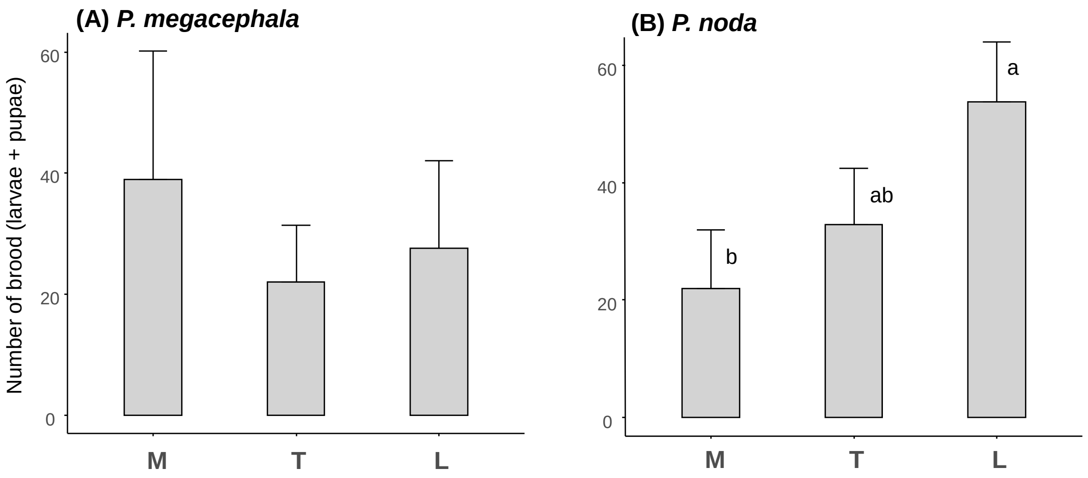

Fig. S1. The total number of brood (larvae + pupae) in **(A)** *Pheidole megacephala* (*N* = 5) and **(B)** *P. noda* (*N* = 5) colonies when ants were provisioned with a food containing one of three protein sources: mealworm (M, control), tryptone (T) and lactalbumin hydrolysate (L). The mean + SE were shown. A statistically significant difference ($p<0.05$) was detected in post-hoc pairwise t tests with pooled SD between treatments with different letters (a and b) after Bonferroni correction.

Effect of protein levels on worker survival in *Pheidole megacephala*

Protein is an essential nutrient for larval growth but is known to be toxic for many adult insects, especially when provided at too high a concentration[1]. To understand whether this was the case in *P. megacephala* and to identify an appropriate protein concentration in the artificial diet, we fed various colonies of this ant for 35 days on six different diets. P1 contained only 14 g pure protein (tryptone) in 100 g of distilled water solution with no other nutrition; diets P2, P3, P4, P5, and P6 had five different protein levels (14, 7, 3.5, 1.4, and 0.7 g of tryptone, respectively), together with a constant per volume amount of the other essential nutrients: lipid (1.4 g), vitamins (1.2 g), minerals (1.2 g), and sucrose (13 g) in 100 g of distilled water solution (for details of those nutrients see the main text). Each diet was hand-mixed well before provisioning. Each experimental colony consisted of one queen and 200 minor workers housed in a small Petri dish (55-mm diameter; similar to the resource distribution experiment). Four colonies were used for each treatment. To assess mortality under each protein level, the number of dead workers in each colony was counted every day for 35 days.

A survival analysis with log rank test was performed in R (v. 3.3.1). Life span of ant workers differed among the six diets ($\chi^2_{(5)} = 847$, $p < 0.001$). Pairwise comparisons revealed that the shortest worker life span occurred with the P1 diet (only-protein vs. any other treatment, $p < 0.001$). With the same high protein concentration but with the other essential nutrients added (P2), worker survival was recovered (P1 vs. P2, $p < 0.001$). However, P2 showed the lowest worker survival among the other mixed diets (vs. each of P3–P6, $p < 0.001$). Among P3–P6 worker longevity was lowest in P3 (vs. each of P4–P6, $p < 0.001$), but there was no significant difference among P4, P5, and P6 (P4 vs. P5: $p = 0.68$; P4 vs. P6: $p = 0.24$; P5 vs. P6: $p = 0.45$). Our result suggests that a high concentration of protein (tryptone, 7 g or more in 100 g of water solution) can be toxic for *P. megacephala* workers. Survival curves of workers in ant colonies fed diets with different levels of protein are illustrated in Figure S2.

Biology Open • Supplementary information

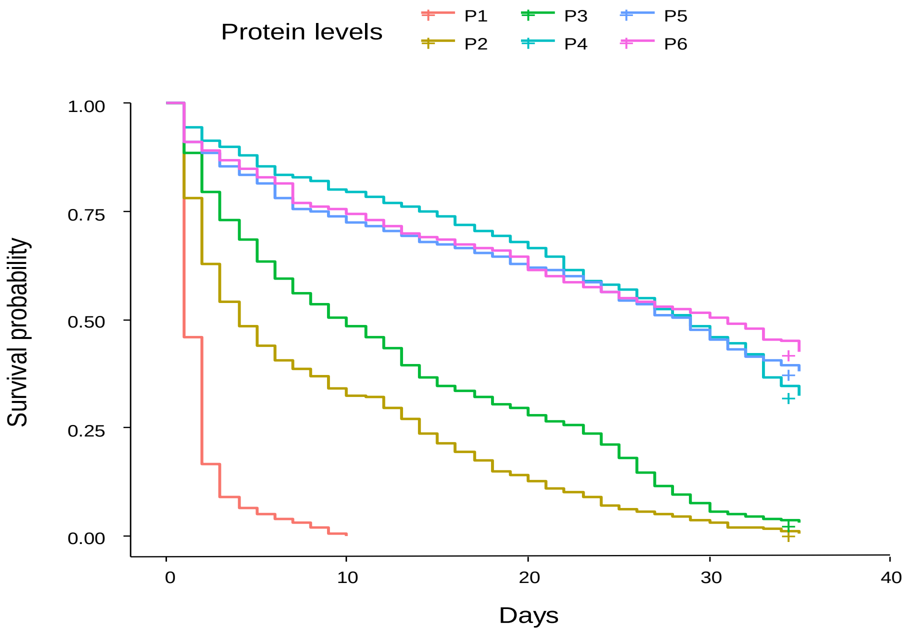

Fig. S2. Worker survival in *Pheidole megacephala* that were fed diets with different protein concentrations. P1 contained 14 g pure protein (tryptone) in 100 g of distilled water solution with no other nutrition. The diets P2, P3, P4, P5, and P6 had five different protein levels (14, 7, 3.5, 1.4, and 0.7 g of tryptone, respectively), together with a constant per volume amount of the other essential nutrients: lipid (1.4 g), vitamins (1.2 g), minerals (1.2 g), and sucrose (13 g) in 100 g of distilled water solution.

Biology Open • Supplementary information
